# Intelligent Detection and Recognition of Marine Plankton by Digital Holography and Deep Learning

**DOI:** 10.3390/s25072325

**Published:** 2025-04-06

**Authors:** Xianfeng Xu, Weilong Luo, Zhanhong Ren, Xinjiu Song

**Affiliations:** College of Science, China University of Petroleum (East China), Qingdao 266580, China; z22090002@s.upc.edu.cn (W.L.); s23090005@s.upc.edu.cn (Z.R.); z23090012@s.upc.edu.cn (X.S.)

**Keywords:** digital holography, marine plankton, deep learning, intelligent detection

## Abstract

The detection, observation, recognition, and statistics of marine plankton are the basis of marine ecological research. In recent years, digital holography has been widely applied to plankton detection and recognition. However, the recording and reconstruction of digital holography require a strictly controlled laboratory environment and time-consuming iterative computation, respectively, which impede its application in marine plankton imaging. In this paper, an intelligent method designed with digital holography and deep learning algorithms is proposed to detect and recognize marine plankton (IDRMP). An accurate integrated A-Unet network is established under the principle of deep learning and trained by digital holograms recorded with publicly available plankton datasets. This method can complete the work of reconstructing and recognizing a variety of plankton organisms stably and efficiently by a single hologram, and a system interface of YOLOv5 that can realize the task of the end-to-end detection of plankton by a single frame is provided. The structural similarities of the images reconstructed by IDRMP are all higher than 0.97, and the average accuracy of the detection of four plankton species, namely, *Appendicularian*, *Chaetognath*, *Echinoderm* and *Hydromedusae,*, reaches 91.0% after using YOLOv5. In optical experiments, typical marine plankton collected from Weifang, China, are employed as samples. For randomly selected samples of Copepods, Tunicates and Polychaetes, the results are ideal and acceptable, and a batch detection function is developed for the learning of the system. Our test and experiment results demonstrate that this method is efficient and accurate for the detection and recognition of numerous plankton within a certain volume of space after they are recorded by digital holography.

## 1. Introduction

As primary producers in nature, marine plankton are not only important food for fish and other economic animals [1] but also biological populations that affect the marine environment. Furthermore, they play a crucial role in the global carbon cycle in ecological balance and safety [2,3]. In particular, their abundance and diversity variation is mostly sensitive to environmental conditions, so their diversity and abundance are always regarded as an effective indicator of environmental and ecosystem stability [4,5]. Harmful algal blooms from plankton groups can cause catastrophic ecological and economic losses [6]. So, the observation of individual plankton species is a key measure to monitor their variation. Exploiting suitable methods to detect marine plankton will benefit ecosystem protection [7,8]. Nevertheless, it is difficult to observe the spatial and temporal variation in their groups by a conventional microscope, not only because of their living environment of seas and oceans but also the strict limitation of the imaging distance, mostly less than one millimeter in a common microscope. It is difficult and time-consuming for humans to adjust the imaging position during the observation process under a conventional microscope. In addition, some plankton and algae are fully transparent, so a white-light microscope is powerless for this kind of phase object. Modern equipment is necessary for plankton group monitoring.

Digital holography (DH) enables complex amplitude (including real amplitude and phase) recording and reconstruction, three-dimensional imaging, no-lens imaging, and non-contact measurements [9,10]. A DH imaging system is a unique imaging system since it leaves out the imaging lens, and its large depth of field makes it suitable for the recording of many objects in a certain space by one single exposure. The advantages above mean that DH is widely used in live cell investigation and plankton detection [11,12]. Although there are many merits in the recording process of DH, there are still some drawbacks in the reconstruction of the original object image. Its off-line setup introduces a tilt reference angle to generate the original object wave, but the contradiction between ensuring that the tilt angle is sufficient to separate the original image and the fact that a small tilt angle is limited by the low resolution of the recording device of CCD hinders its application. The in-line setup of phase shifting DH (PSDH) solves this contradiction, but it needs more than at least two frames to calculate the object wave, which makes it time-consuming. In addition, DH needs a tightly controlled laboratory environment to ensure the hologram quality, which makes it unsuitable for plankton imaging in water. Moreover, the inconveniences of the DH method also include its computationally expensive reconstruction and coherent noise associated with laser interferences. All of these factors can make the post-processing of holographic imaging challenging [13].

Fortunately, advances in computational hardware and the development of algorithms boost the application of biological imaging and classification. In the deep learning (DL) method, convolutional neural networks (CNNs) have been suggested for plankton imaging and identification. For example, Liu et al. evaluated several models, AlexNet, VGG16, GoogleNet, PyramidNet, and ResNet, and then pointed out that PyramidNe can improve the accuracy of the Woods Hole Oceanographic Institution (WHOI) Plankton dataset [14]. However, these machine learning plankton detection and classification methods cannot be directly applied to holographic data because holographic patterns are morphologically different from microscopic images. Some research groups have recently developed some deep learning-based methods for processing plankton holograms.

Bachimanchi et al. used recorded plankton holograms to segment planktons from a background by using Regression U-Net (RU-net) and then detailed their rough locations [15]. Cotter E et al. used a deep learning network to select suitable holograms for automated processing and then detected the diffraction patterns of targets in the original holograms and reconstructed these targets by using a small window around the diffraction patterns further. Evidently, this deep learning method provides a significant reduction in computational cost and data storage requirements [16].

In this paper, an intelligent method for the detection and recognition of marine plankton (IDRMP) is proposed by using DH and DL. We first simulate a digital hologram of plankton by the recording principle of digital holography. There are four kinds of plankton, namely, *Appendicularian*, *Chaetognath*, *Echinoderm*, and *Hydromedusae,*, that we employ, and the number and position of the four kinds of plankton are randomized in the holograms. Subsequently, the simulated holograms and the images reconstructed by the high-quality two-step PSDH algorithm are divided into two groups of training sets and test sets, and both of them are used to train the designed A-Unet model to complete the image reconstruction of the plankton with a single hologram. Then, the reconstructed plankton images and the corresponding plankton labels are put into the YOLOv5 network for training to complete the recognition of plankton. Finally, optical experiments are carried out for verification. Seawater plankton obtained from Weifang, China, are used as samples in the experiments for the detection of three kinds of plankton including Copepods, Tunicates, and Polychaetes. By using PyQt5 to design the system interface, an end-to-end integrated system for the reconstruction and recognition of plankton images is realized, and then a batch detection function is developed. The test results of the IDRMP show that the system proposed here can detect and recognize a large number of plankton recorded in one DH hologram with high accuracy, which is very important for humans to explore the ocean, to monitor ecological health, to prevent disasters, and to establish seawater farms. In Section 2, the principles of DH recording and reconstruction are introduced and the method used to train the deep learning network is also explained. The deep learning structure is designed in Section 3, with Section 4 introducing the specific recognition method. Then, Section 5 presents the optical experiment verification and the end-to-end integrated system, followed by the summary.

## 2. Digital Holography and Dataset Production

Here, the in-line PSDH method is used to generate holograms and reconstruct the object image for deep learning network training.

### 2.1. Recording of Digital Hologram

**Figure 1:**
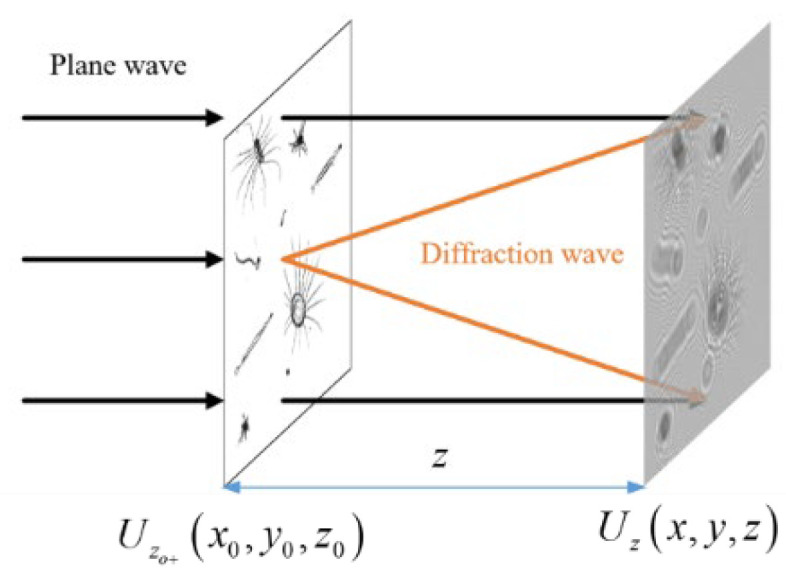
Digital Hologram Recording.

For coaxial DH (see Figure 1), where both the reference and object waves are on the same optical axis, an object can be described by a transmission function *o* (*x_o_*,*y_o_*) on a given plane [17]:

(1) $$o\left(x_{o},y_{o}\right)=\exp\left[-a\left(x_{o},y_{o}\right)\right]\exp\left[i\phi \left(x_{o},y_{o}\right)\right]$$

where *a* (*x_o_*,*y_o_*) represents the absorption coefficient of the object; *ϕ* (*x_o_,y_o_*) is the phase distribution. The wavefronts *Uz_o_*_+_ (*x_o_*,*y_o_*) behind the object can be calculated from the transmission function as follows:

(2) $$U_{z_{o}+}\left(x_{o},y_{o}\right)=o\left(x_{o},y_{o}\right)U_{z_{o}-}\left(x_{o},y_{o}\right)$$

where *Uz_o_*_−_ (*x_o_*,*y_o_*) is the incident wave, which is a plane wave mostly. The outgoing wave propagates along the optical axis towards the recording plane located at *z*. This can be approximated by the Fresnel diffraction, as shown below:

(3) $$U_{z}\left(x,y\right)=\frac{\exp\left(ikz\right)}{i\lambda z}\iint U_{z_{o}+}\left(x_{o},y_{o}\right)\exp\left[ik\frac{{\left(x-x_{o}\right)}^{2}+{\left(y-y_{o}\right)}^{2}}{2z}\right]dx_{o}dy_{o}$$

where *λ* is the wavelength, *k* = 2π/*λ* is the wave number, and *z* is the distance between the object plane and the recording plane.

If the reference wave is a plane wave with a relative phase to the object wave of a constant *δ* and the real amplitude of a real constant *A_r_*, the plane wave of the reference can be expressed as follows:

(4) $$U_{r}=A_{r}\exp\left[i\delta \right]$$

After the interference of the object wave and the reference wave, the interference intensity distribution on the recording plane is as follows:

(5) $$I\left(x,y\right)=\left[U_{z}\left(x,y\right)+U_{r}\right]{\left[U_{z}\left(x,y\right)+U_{r}\right]}^{*}$$

where the symbol “*” is the complex conjugate. Once *λ*, *z*, *Uz_o_*_−_ (*x_o_*,*y_o_*) and *o* (*x_o_*,*y_o_*) are known or set, the hologram can be simulated. In PSDH, several different holograms are generated by changing the reference phase *δ* (*x*, *y*) in Formula (4).

### 2.2. Reproduction

In PSDH, the phase shift value is measured in experiments and then the object wave on the recoding plane is calculated. Inverse Fresnel diffraction is simulated to complete the reconstruction of the original object wave on the original object plane. In 2007, L. Cai [18] et al. proposed generalized phase-shift digital holography (GPSDH), which no longer requires precise phase-shift control in experiments but extracts the phase-shift values from the interferograms for better practicality. In this method, a high-quality two-step GPSDH is used to reconstruct the object image.

With the coordinates (*x*, *y*) omitted for clarity in the following section, the wavefront of the object wave and the plane wave of reference on the recording plane can be denoted as follows:

(6) $$U_{o}=A_{o}\exp\left[-j\varphi_{o}\right]$$

(7) $$U_{r}=A_{r}\exp\left[-j\delta_{i}\right],i=0,1,2,3\cdots$$

where *A_o_* and *φ_o_* are the real amplitude and phase of the object wave, *A_r_* is the reference amplitude, and *δ_i_* denotes the relative phase value of the reference wave corresponding to the *i*th hologram. For two-step GPSDH, if the reference phase of the first hologram is 0 and the phase difference between the two adjacent references of holograms is *δ*, the holograms can be expressed as follows:

(8) $$I_{1}=A_{o}^{2}+A_{r}^{2}+2A_{r}A_{o}\cos\varphi_{o}$$

(9) $$I_{2}=A_{o}^{2}+A_{r}^{2}+2A_{r}A_{o}\cos\left(\varphi_{o}-\delta \right)$$

By using Equation (8) and Equation (9), Equation (10) can be obtained as follows:

(10) $$\sin\varphi_{o}=\frac{I_{2}-I_{1}}{2A_{o}A_{r}\sin\delta }+\frac{\left(I_{1}-I_{o}-I_{r}\right)\tan\frac{\delta }{2}}{2A_{r}A_{o}}$$

After using Euler’s formula for Equations (8) and (10), the object wave can be rewritten as follows:

(11) $$U_{o}=\frac{I_{1}-I_{0}-I_{r}}{2A_{r}}+i\frac{I_{2}-I_{1}\cos\delta -\left(1-\cos\delta \right)\left(I_{o}+I_{r}\right)}{2A_{r}\sin\delta }$$

Equation (11) shows that the reconstruction of the object wave requires the phase shift value and four intensity maps, including the intensity map of the object wave and the reference wave, and two interferograms.

### 2.3. Dataset Generation

**Figure 2:**
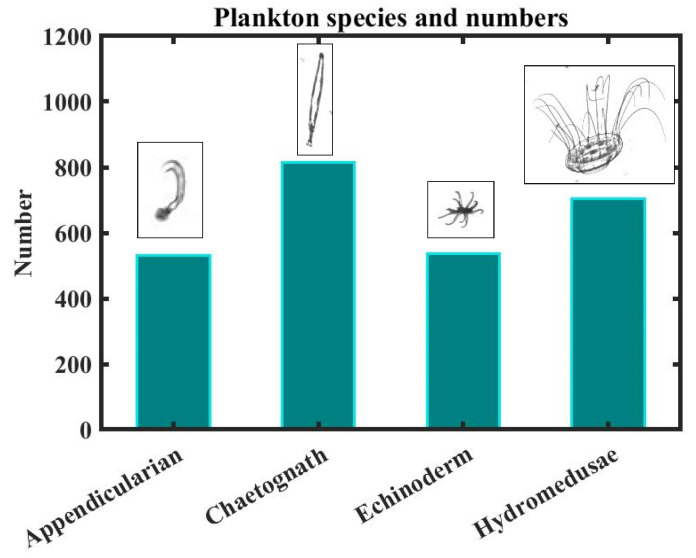
Plankton species and their numbers used for simulations.

In order to generate datasets that can be used for target segmentation and detection tasks, the transmission function *o* (*x_o_*,*y_o_*) of several targets in the plane *z_o_* needs to be established. A composition of shadow images is collected by the In Situ Fish-Plankton Imaging System (ISIIS), the subject of a competition on Kaggle (a big data competition platform) [19]. This open-source dataset consists of 121 species of marine plankton, of which 4 species with a number of images greater than 500 were selected for our simulations, as shown in Figure 2.

Pure amplitude objects are considered for plankton detection and the detection process involves extracting seawater to produce areas of *ϕ* (*x_o_*,*y_o_*) = 0. The simulation process for *o* (*x_o_*,*y_o_*) is as follows. Firstly, for each *o* (*x_o_*,*y_o_*), 5–10 plankton images are randomly selected among all the four species of plankton images. Then, these images were randomly rotated in four possible ways (0°, 90°,180°,270°) and flipped in three possible ways (none, horizontal or vertical). Then, 5–10 transmission functions with no overlap are assigned on a 512 × 512 empty frame to generate *o*(*x_o_*,*y_o_*), and the bounding box corresponding to each plankton picture is simultaneously derived as a label for the target recognition network. Finally, holograms *I* (*x*,*y*) were generated by using Equations (3), (4) and (5). All of the holograms are normalized by their maximum values.

**Figure 3:**
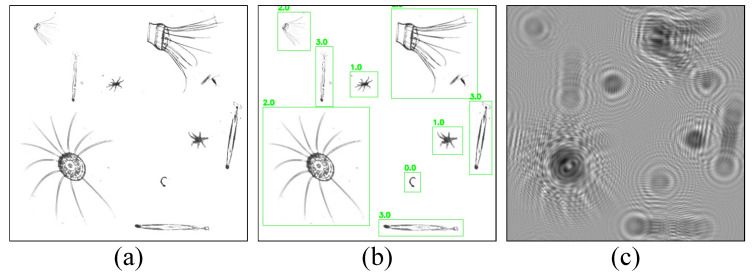
Plankton images and hologram: (**a**) original image; (**b**) YOLO labeled images; (**c**) hologram.

In the simulation, the laser wavelength is *λ* = 530 nm, the recording distance is *z* = 24 cm, and the incident plane wave is set as *Uz_o_*_−_(*x_o_*,*y_o_*) = 1. The resolution of the recording device is 512 × 512 with each pixel size of 5 μm × 5 μm. In our study, 9000 and 1000 holograms were simulated for training and testing, respectively. Figure 3 shows an example of the simulated and labeled *o* (*x_o_*,*y_o_*) with their corresponding holograms. In Figure 3b, the numbers 0.0, 1.0, 2.0, and 3.0 above each green box represent plankton of *Appendicularian*, *Echinoderm*, *Hydromedusae,* and *Chaetognath*, respectively.

## 3. Holography Reconstruction Model

**Figure 4:**
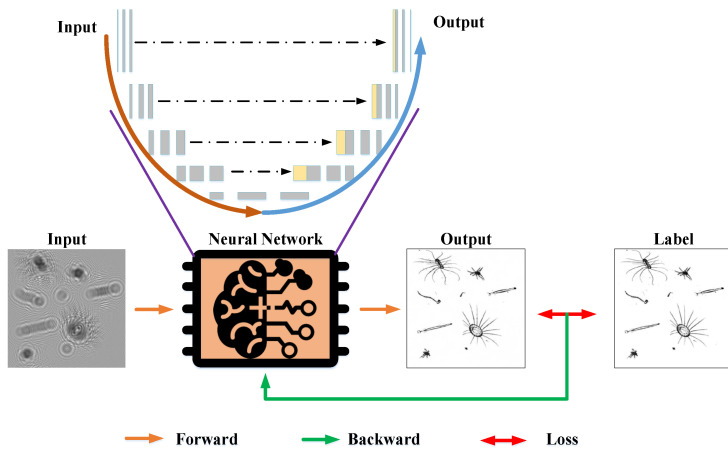
DL process of the neural network.

In nature, the deep learning method includes a training procedure to develop a network by employing the existing data packet so that the network can work as an optimized matching system in application. Its learning mechanism consists of two critical loops: in the feedforward computation, input samples undergo multilayer nonlinear transformations to generate predictive outputs; by comparing the pixel-level differences between these predictions and the annotated data (as illustrated in Figure 4, where the small differences between the output image and the labeled image are more obvious when the figure is enlarged), a loss function is constructed and error backpropagation is performed [20]. During this process, the loss gradients guide the parameter update strategy, iteratively optimizing the network to approach the global and local optimum.

It is noteworthy that the quality of the dataset determines the biological upper limit of model performance. Research indicates that the training of samples with high diversity and low redundancy enables the network to capture richer pattern representations, thereby a more robust distribution in the parameter space can be achieved. This synergistic evolution of data and models stands as a pivotal cornerstone in the groundbreaking advancements of contemporary deep learning.

### 3.1. Holographic Reconstruction Network

**Figure 5:**
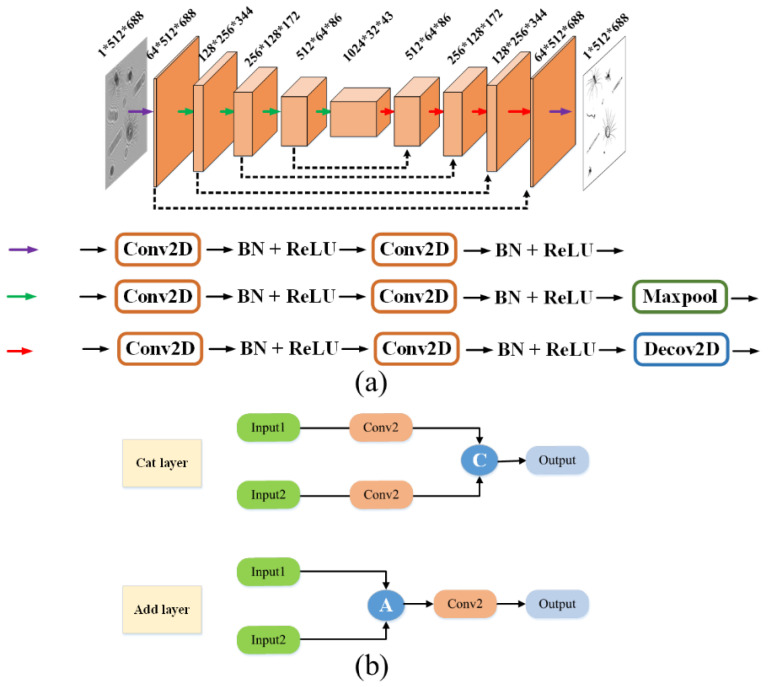
Model structure of A-Unet network: (**a**) framework of the model; (**b**) difference between add and cat layers.

For the task of object reconstruction by a single hologram frame, an improved A-Unet model is proposed on the classical basic U-net architecture. As shown in Figure 5, the network adopts a symmetric encoder–decoder structure, comprising two core components of the down-sampling encoding path and the up-sampling decoding path. It establishes cross-hierarchical information transmission channels through symmetrically distributed residual mapping modules. Three feature operation units are defined during the model construction process. The purple basic feature extraction unit, composed of a two-dimensional convolutional layer, batch normalization operation [21], and ReLU activation operation, achieves the layered extraction and nonlinear representation of multi-scale structural features. Based on the purple unit, the green down-sampling unit, incorporating a max pooling layer, builds a hierarchical abstraction mechanism to enhance the key feature responses through spatial dimension compression. Equipped with a transposed convolutional layer (de-convolutional layer), the red up-sampling unit replaces the fixed interpolation algorithms with learnable deconvolution kernels to significantly improve the restoration accuracy of the high-frequency details during the feature reconstruction process. The symmetrically distributed residual connections between the encoding and decoding paths not only preserve the multi-resolution feature fusion capability of the original U-net but also effectively alleviate information degradation issues in network training by establishing gradient propagation shortcuts. This enables the model to achieve the synergistic optimization of reconstruction accuracy and generalization performance while maintaining lightweight characteristics.

The loss function used in the model is the mean square error (MSE Loss). For the hologram reconstruction problem, our experimental results indicate that taking the mean square error function as the loss function results in a faster optimization convergence speed. The MSE (Mean Squared Error) function is primarily used to directly optimize the pixel-level numerical matching between the reconstructed image and the real image. It solves the deep learning-generated image against the label image and then optimizes the network by backpropagation. The smaller the loss value, the closer the network is to the optimal solution. The Adam optimizer [22] was chosen for the model optimizer here. The optimization algorithm with an adaptive learning rate clearly has a faster optimization speed and the convergence can be improved by controlling the Adam learning rate [23].

In the U-net model, the feature fusion module in the up-sampling uses a cat layer, where each feature is first passed through a convolutional layer, followed by the fusion of each feature according to different weights. In the designed A-Unet model, the cat layer is replaced with the add layer. The feature maps from the down-sampling stage that bypass the two convolutional layers are directly connected to the feature maps in the up-sampling stage for fusion through residual addition. The first letter “a” in the word “addition” is used as the prefix “A” in “A-Unet”. For all the incoming features, all of the features are first superimposed, and after superimposition, they go through a convolutional layer to compute the weights. The model structure is shown in Figure 5a. As shown in Figure 5b, the add layer can reduce the parameters, and then greatly improve the speed of the network and prevent overfitting, which makes the network only applicable to the training set.

**Figure 6:**
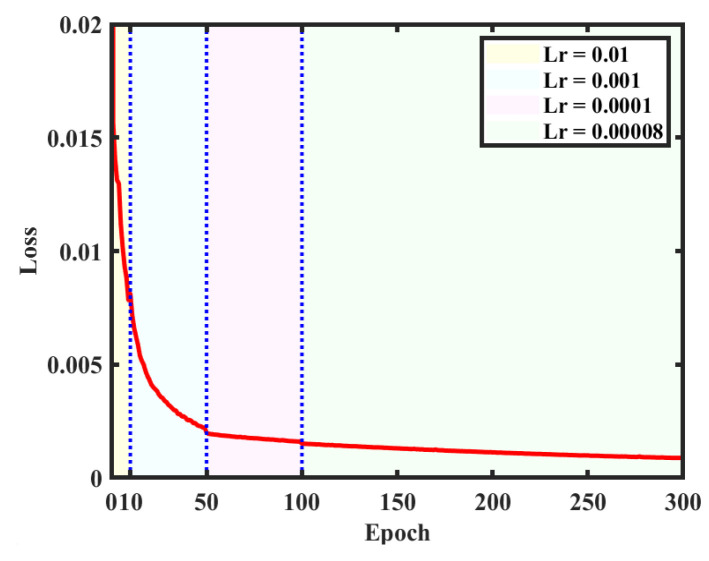
Loss value variation with training cycles.

The created dataset is used for training on the structural parameters of the network. There are 10,000 digital holograms and corresponding reconstructed object images as labels in the dataset. After inputting holograms into the network, the reconstructed object images pass through the network forward and are compared with the labels for model correction. Among them, 9000 are used for training and 1000 are employed for testing. In order to achieve faster convergence, the learning rate (Lr) is set to 0.01 and the network is trained for 10 cycles at the beginning. Then, a learning rate of 0.001 is set for the next 40 cycles and a learning rate of 0.0001 is used for another 50 cycles. When the loss values stabilize, a learning rate of 0.00008 is finally used for 200 cycles of training to optimize the network weights. The curve of the loss value is given in Figure 6, where different colored backgrounds correspond to different learning rates. It can be observed that the training results after 300 cycles gradually reach the optimal solution (or local optimal solution), so the training is stopped after 300 cycles. The GPU used for training is RTX3060, with a total time of 57.1 h.

### 3.2. Analysis for the Training Results of the Reconstruction Network

**Figure 7:**
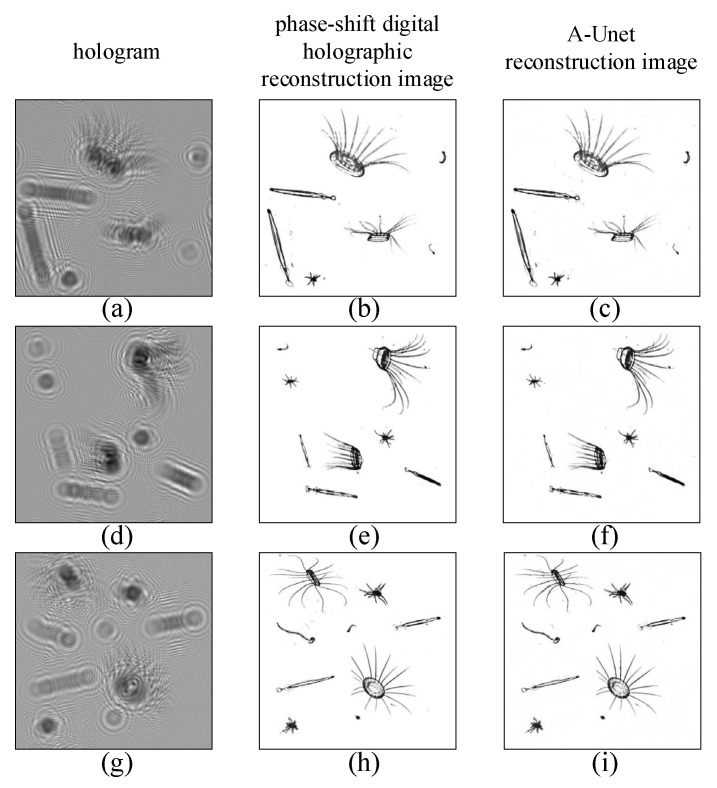
Reconstruction results by GPSDH and DL methods. (**a**,**d**,**g**) hologram; (**b**,**e**,**h**) phase-shift digital holographic reconstruction image; (**c**,**f**,**i**) A-Unet reconstruction image.

In Figure 7, the reconstructed image by two-step GPSDH is compared with that from the deep learning model. Figure 7a,d,g show the holograms, Figure 7b,e,h are the corresponding images reconstructed by GPSDH, and Figure 7c,f,i represent the images reconstructed by DL, respectively. The results in Figure 7 show that the A-Unet network designed here is capable of achieving acceptable reconstruction of plankton images, laying a solid foundation for the subsequent recognition tasks.

In DL, model stability is an important indicator to ensure its practical value. For the A-Unet reconstruction network, judging the similarity between the reconstructed image by the DL model and that from the GPSDH is the core criterion for verifying the accuracy of the network.

In the assessment of imaging quality by DL, the Structural Similarity Index (SSIM) [24] is one of the primary methods in evaluating the pixel-level errors and the mismatch between visual perceptions of hologram reconstruction tasks. SSIM assesses the luminance, contrast, and structure of two images, providing an objective evaluation of the differences between images reconstructed by DL methods and the label images reconstructed by GPSDH algorithms.

The SSIM function can be expressed as follows:

(12) $$\mathrm{SSIM}(x,y)=\frac{\left(2\mu_{x}\mu_{y}+c_{1})(2\sigma_{xy}+c_{2}\right)}{\left(\mu_{x}^{2}+\mu_{y}^{2}+c_{1})(\sigma_{x}^{2}+\sigma_{y}^{2}+c_{2}\right)}$$

In Equation (12), *x* and *y* represent the two normalized images to be obtained, respectively, *μ_x_* and *μ_y_* are their mean values, *σ_x_* and *σ_y_* are the standard deviations for them, *σ_xy_* is the covariance of *x* and *y*, and *c*_1_ = (*k*_1_*L*)^2^, *c*_2_ = (*k*_2_*L*)^2^ are two constants with *k*_1_ = 0.01, *k*_2_ = 0.03. For an 8-bit binary image, *L* = 255. The values of SSIM are distributed in the range of (0, 1). The closer the value is to 1, the higher the similarity. The SSIM value of two identical images is 1.

**Figure 8:**
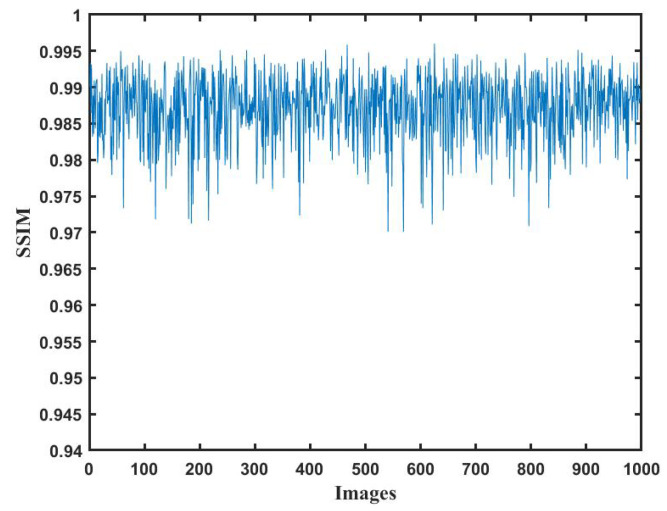
SSIM from the images in the test.

The variation curve of the reconstruction quality reflects the performance of the network for different holograms. The SSIM values between the images reconstructed by DL and the labeled image are shown in Figure 8. It can be seen that the structural similarities in the test set are all higher than 0.97. The A-Unet network can be competent in reconstructing the object image from digital holograms.

## 4. The Plankton Recognition Network

### 4.1. YOLOv5 Network

The original YOLO model for target detection was developed by Joseph et al. in 2016 [25]. Many researchers have subsequently improved this model [26]. The model used in this paper is YOLOv5 [27], an algorithm obtained by Glenn Jocher. On the basis of YOLOv4, the network was optimized to YOLOv5, which has a faster speed for target detection compared to YOLOv4 and better detection accuracy for small targets.

The YOLO series networks make the detection a regression problem. After the images are input and go through a neural network, YOLO can directly obtain both the bounding box and the corresponding probability of this bounding box belonging to the category. Because the entire detection process uses only one network, it can realize the end-to-end (one end means the input of the raw data, the other end is the output of the final result, and all the feature learning operations are assembled into the network, without separate processing) optimization.

**Figure 9:**
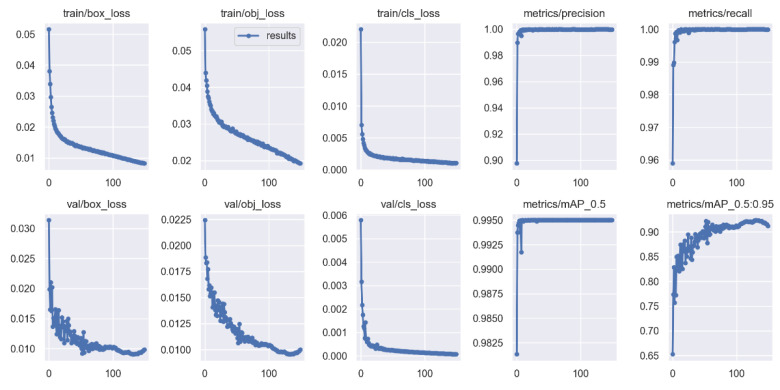
Loss variation from YOLOv5 model.

Our plankton recognition method uses the YOLOv5 model of the YOLO family. In total, 10,000 digital holographic reconstruction maps of plankton are divided into a training set and testing set according to a 9:1 rate, and the loss value of the training process is shown in Figure 9.

From Figure 9, it can be found that the loss values in both the training process and the validation process gradually decrease as the training cycle advances, leaning towards the lowest value when it reaches 100 cycles, and the mean accuracy percentage (mAP) [28] is higher than 95%.

**Figure 10:**
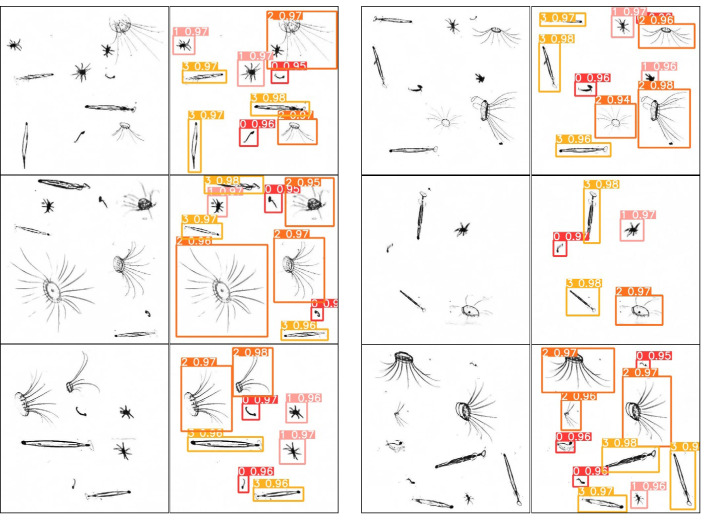
Plankton recognition by YOLOv5 model.

The model was then validated using the plankton map, and some of the results are shown in Figure 10. In the second and fourth columns, the numbers 0, 1, 2, and 3 above each box represent plankton of *Appendicularian*, *Echinoderm*, *Hydromedusae*, and *Chaetognath*, respectively, and the other decimal number behind each of them is the confidence level, which indicates the possibility of the detection box containing the target.

### 4.2. Analysis of YOLOv5 Recognition Results

In the training process, the plankton samples are divided into four categories. For simplicity, the numbers 0, 1, 2, and 3 are used instead of the names of the species plankton category, respectively. All the validation images can be recognized here by our YOLOv5 network.

In the network for target detection, there are four concepts, which are listed below:

①True positives (TPs) mean positive samples that are correctly recognized as positive samples;

②True negatives (TNs) mean negative samples that are correctly recognized as negative samples;

③False positives (FPs) mean false positive samples, i.e., negative samples that are incorrectly recognized as positive samples;

④False negatives (FNs) mean false negative samples, i.e., positive samples that are incorrectly recognized as negative samples.

Precision, also called checking accuracy, refers to the percentage of samples that are predicted to be positive out of all the samples that are indeed positive. In general, the higher the accuracy, the better the classifier. When FP = 0, the accuracy is 100%.

Recall, also known as the check-all rate, refers to the percentage of samples that are indeed positive out of all the samples that are predicted to be positive. The larger the Recall, the better. When FP = 0, the target can be detected with 100% possibility.

**Figure 11:**
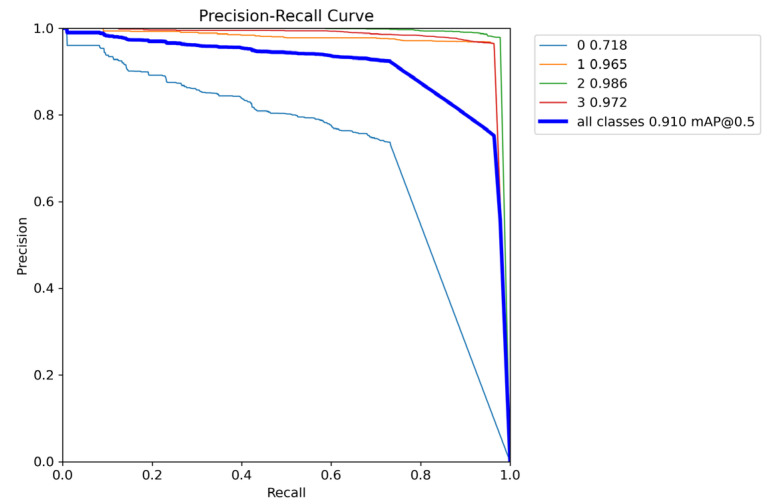
Relationship between precision and recall.

The precision–recall (PR) curve is the detection accuracy–detection rate. When the PR curve is closer to the upper right, it indicates that the performance of the model is better. The PR curve of the plankton by our biometric model is shown in Figure 11:

In Figure 11, the YOLO model we trained is tested at a confidence threshold of 0.5, achieving an overall accuracy of 91.0%. Here, numbers “1”, “2”, and “3” represent *Echinoderm*, *Chaetognath*, and *Hydromedusae,*, respectively, with a total precision of over 96%. “0” represents *Appendicularian*. Although it can be recognized, the precision of the bounding box is only 71.8%. This is because Appendicularia are relatively small targets, and the symbol mAP indicates that a prediction is considered correct when the overlap ratio between the predicted box and the ground truth box exceeds 0.5. Therefore, although some predicted boxes may have a smaller overlap area, it does not affect the identification of the plankton species.

## 5. Experimental Verification and Integrated System Design

After completing the model construction and numerical simulation, optical experiment validation is carried out. The samples for the experiments were taken from seawater in the Weifang coastal area of China and underwent basic filtration to ensure their quality.

**Figure 12:**
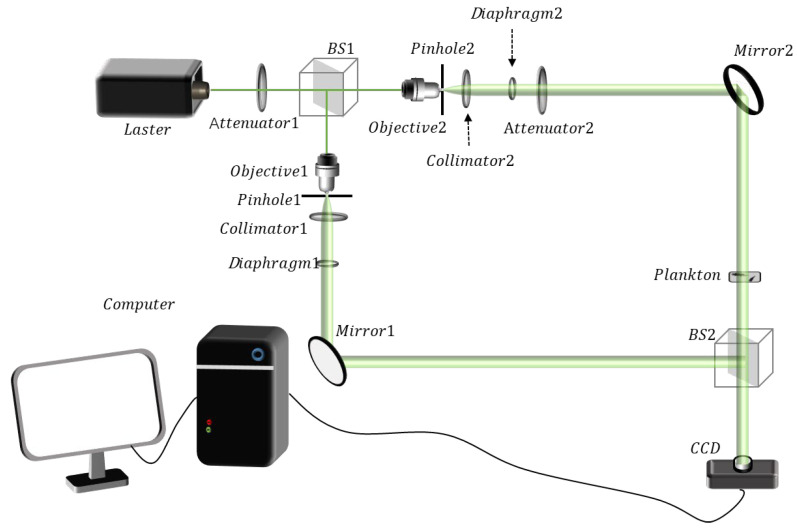
Holographic recording diagram of marine plankton.

The experimental setup is shown in Figure 12. A green laser (MSL-FN-532, produced by CNI from Changchun, China) is adopted as the light source. The laser beam is split into two by a beam splitter. Then, the two beams pass through the objective lens, pinhole filtering, and a collimating lens, respectively, to obtain high-quality uniform plane waves. One of the beams serves as the reference wave, while the other is used as the object wave to irradiate the plankton samples and trigger the diffraction effect. The diffractive wave carries the detailed structural information of the samples. Subsequently, it overlaps spatially with the reference wave through a beam combiner, thus generating a hologram, which is then recorded with high fidelity by a CCD (DH-SV1410FM, produced by IMAVISION from Beijing, China). During the process of the experiment, the phase shifts are effectively generated by disturbances such as air disturbance and vibrations from optical devices.

**Figure 13:**
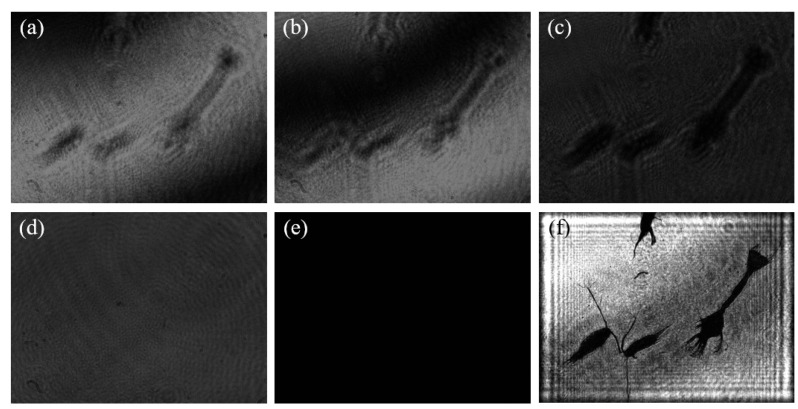
Results obtained by GPSDH: (**a**,**b**) holograms captured by CCD; (**c**) object wave; (**d**) reference wave; (**e**) background; (**f**) reconstructed image.

A set of plankton sample data obtained by the two-step GPSDH technique is presented in Figure 13. Specifically, (a) and (b) display two interference patterns required by the reconstruction algorithm, and these interference patterns can be obtained simply by introducing phase shift [18]. The phase difference between the two holograms is 1.7902 radians by GPSDH calculation. Figure 13c,d,e are the object wave, the reference wave, and the background, respectively. They are needed in the reconstructed process of the two-step algorithm. Finally, the image shown in (f) is the reconstructed image of the plankton sample by two-step GPSDH, which clearly demonstrates the morphological and structural characteristics of the sample.

In this experiment, a total of 4000 holographic images of plankton are collected, and GPSDH is used to perform object image reconstruction. These reconstructed images are then used as the label images in the training process of the A-Unet network to ensure that the network could accurately reconstruct the holographic images of plankton. Each of the original images have a size of 1392 × 1040 pixels, providing abundant detail information for subsequent analysis.

Then, the dataset is divided into a training set and a testing set at a ratio of 9:1. This processing procedure not only enhances the feasibility of the experiment but also provides a high-quality data foundation for the subsequent training of the A-Unet network.

**Figure 14:**
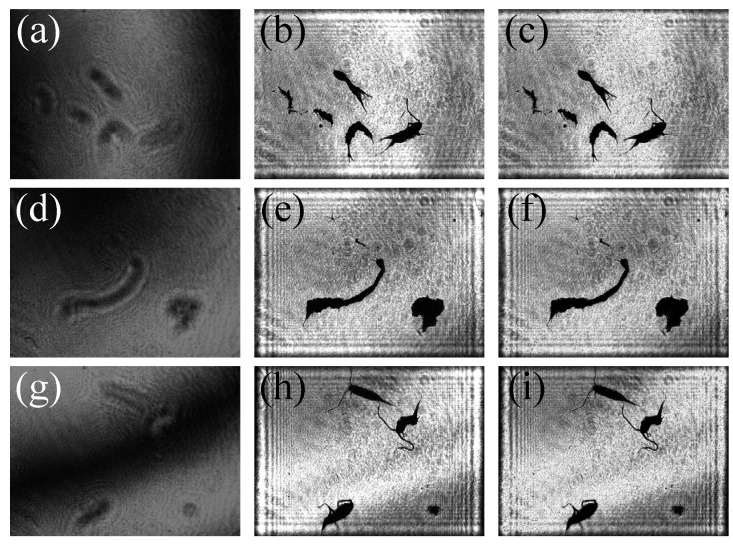
Imaging results obtained by different methods: (**a**,**d**,**g**) holograms; (**b**,**e**,**h**) results obtained by the GPSDH; (**c**,**f**,**i**) results obtained by IDRMP.

After 16.5 h of training, the trained deep learning model is applied on the test set for evaluation. Figure 14 shows the comparative analysis between the images reconstructed by GPSDH and those reconstructed by DL techniques. Figure 14a,d,g display the originally collected hologram of the samples; Figure 14b,e,h present the images reconstructed by GPSDH; and Figure 14c,f,i are the image results reconstructed by using the IDRMP. From the comparison and analysis, it can be found that a small number of tiny black dots appear in the background of the images reconstructed by the DL model. This phenomenon is caused by the noise in the holograms, which does not affect the subsequent identification and detection. According to the details in plankton images, the DL model demonstrates extremely high precision, and its performance shows no difference compared with that of traditional algorithms, which usually take a relatively long time. The two-step GPSDH algorithm takes an average of 0.9376 s to create high-quality object images with the input of 5 required images, while the A-Unet takes an average of only 0.0720 s on average to reconstruct object images. This result fully demonstrates the great potential of the IDRMP method in the field of plankton detection.

**Figure 15:**
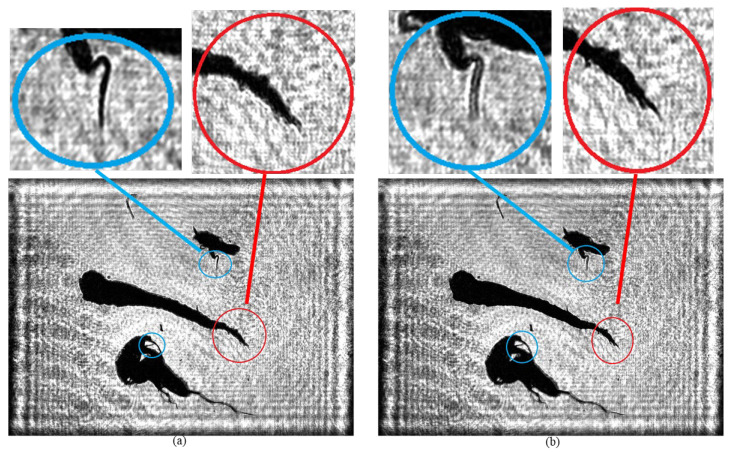
Plankton images on different focal planes: (**a**) imaging at a distance of 100 mm from the recording plane; (**b**) imaging at a distance of 102 mm from the recording plane.

To completely preserve the tissue structure and morphological characteristics of plankton, an environment with a seawater depth of 2000 μm is usually employed. Given that the thickness of plankton is generally from 50 to 300 μm, this condition can not only effectively avoid causing damage to plankton but can also significantly reduce the occurrence probability of overlapping among organisms. However, when using DH for imaging, since different plankton are distributed on planes with different depths, it is difficult to simultaneously focus on all plankton clearly in one image. Specifically, when focusing on the plankton on the plane with a specific depth, the images of plankton located on other planes will inevitably be blurred to some extent, as shown in Figure 15.

As shown in Figure 15, there are significant differences in the imaging clarity of plankton on different planes when using traditional holographic algorithms. In Figure 15a, the branch and angle structures of the two plankton marked by the blue circle are clearly visible, while the tail branches and angles of the Tunicate plankton within the red circle are difficult to identify. In contrast, in Figure 15b, the tail of the Tunicate plankton within the red circle shows clear bifurcation and protrusion characteristics, but the branch and angle structures of the two plankton within the blue circle are somewhat blurred.

**Figure 16:**
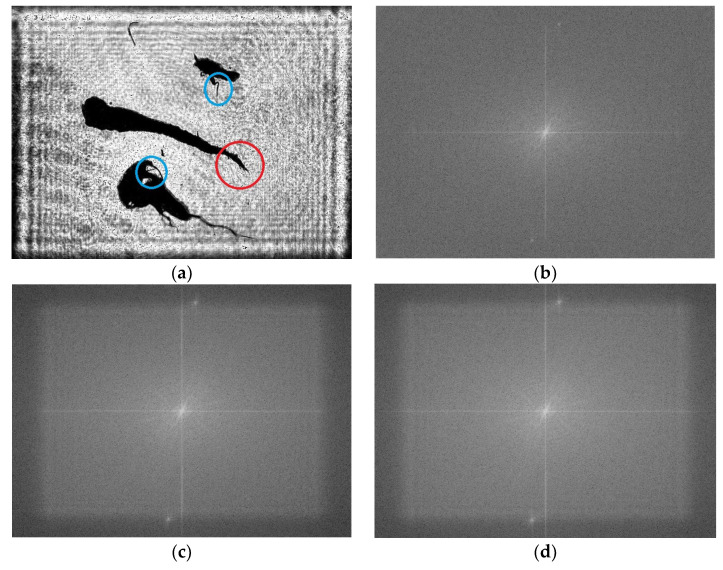
Results obtained by DL and the spectra for the reconstructed images: (**a**) plankton image from DL method; (**b**–**d**) the Fourier spectra of Figure 15a,b and Figure 16a.

In this study, the recording distances for the 4000 holograms of plankton samples are randomly set from 100 mm to 105 mm to ensure the diversity and randomness of the dataset. Through the trained A-Unet network model, precise reconstruction of a single holographic image can be achieved, enabling plankton originally on different planes to obtain a clear focusing effect simultaneously in the same image. As shown in Figure 16, the bifurcation and protrusion structures of the tail of the Tunicate in the central area are presented with high clarity (marked by the red circle frame). Meanwhile, the branch and angle parts of the plankton also maintain extremely high clarity without any blurring (marked by the blue circle frame).

To analyze further the qualities of the images reconstructed by GPSDH and DL, Fourier transform operations are carried out, as shown in Figure 15a,b and Figure 16a, respectively. Their corresponding spectrum distributions in the spatial frequency domain are shown in Figure 16b–d. Since the DL method uses convolution to extract and synthesize the features of images in theory, the resolution of the images reconstructed by DL always degrades. However, the differences among Figure 16b–d are not obvious, showing that the quality of the image from DL can be guaranteed in resolution. It seems that the spectrum in 16b is more continuous and smooth, with some high frequency components from image noise removed. These spectrum distributions show that the images reconstructed are beneficial not only for human observation but also for plankton detection and recognition.

After experimental verification and model testing, the A-Unet designed in this study successfully achieved the goal of reconstructing high-quality images of plankton on planes with different depths through a single holographic image. This system can accurately focus on plankton on different planes in the same image. This characteristic not only promotes the accuracy of the subsequent identification and detection but also effectively avoids the problem of data redundancy caused by collecting multiple images.

Subsequently, the LabImg ( LabIag 3.0) software is used to label the images reconstructed from the 4000 plankton holograms. The labeling categories include four types of plankton: Copepods (labeled as “0”), Tunicates (labeled as “1”), Polychaetes (labeled as “2”), and other types of plankton with non-target particulate matter (labeled as “3”). After the labeling is completed, the dataset is divided into a training set and a testing set at a ratio of 9:1 and they are input into the YOLOv5 network for training. After 100 training epochs, the success rate of the model’s recognition categories reaches 100%, and the precision rate of the prediction box size reaches 90%.

In order to achieve more convenient and intelligent human–computer interaction, PyQt5 is rescued to design the DL into an end-to-end system. PyQt5 5.15.10 is the Python binding for Qt5, which provides comprehensive support for Qt5 library, enabling DLto develop graphical user interface applications in a multi-platform environment on the Python platform [29]. By PyQt5, applications similar to those on the C++ platform can be efficiently designed and implemented by using Python and then be deployed quickly and accurately to various platforms. Based on this framework, an integrated system for plankton detection and identification is developed and the implementation process of the algorithms is visualized.

**Figure 17:**
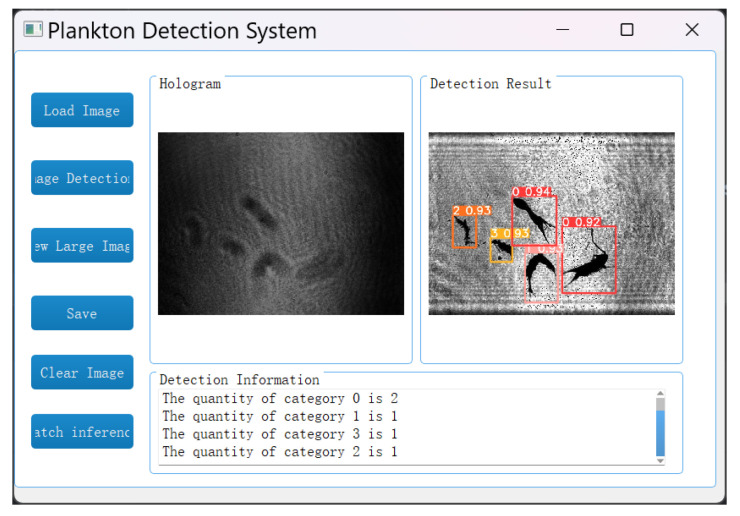
Plankton detection system.

In the image detection system, the reconstruction and identification of holograms is achieved by loading the holograms of plankton and selecting the objects to detect. Specifically, for the holograms of marine plankton, the A-Unet network is first applied to reconstruct the object images and then the YOLOv5 network is used for target recognition and detection. Eventually, the detection results are presented in the form of images, and the quantity statistics of each type of plankton are provided, as shown in Figure 17. The average reconstruction time for each image is 0.0130 s, the recognition time is 0.0147 s, and the total processing time is only 0.0277 s. The computer configuration used in this study includes a Core i7–12700H CPU and an RTX 3060 GPU.

**Figure 18:**
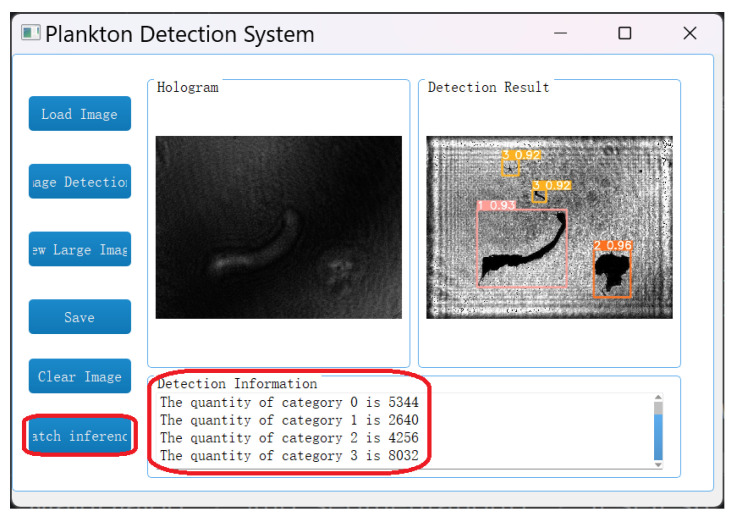
Batch detection results from the plankton detection system.

To better meet the requirements of practical applications, a batch detection function for this system is developed. As shown in Figure 18, by clicking the “batch inference” button, users can select a folder containing any number of plankton holograms. The system will quickly identify all the holograms in the folder and display the types and quantities of each type of plankton. Taking this experiment as an example, a folder containing 4000 plankton holograms is selected. The batch detection function can identify 5344 Copepods (labeled as “0”), 2640 Tunicates (labeled as “1”), 4256 Polychaetes (labeled as “2”), and 8032 other types of plankton with non-target particulate matter (labeled as “3”).

## 6. Summary

The IDRMP method proposed by us can solve the difficult problem of reconstructing object images from the digital holograms of plankton by constructing a DL network model for the reconstruction and recognition. The designed A-Unet is used to carry out the reconstruction by a single hologram containing the information of multiple plankton individuals. A total of 10,000 sets of digital holograms synthesized randomly from plankton organisms are used as the dataset, and the structural similarity of all the reconstruction results is higher than 0.97. In addition, the YOLOv5 model is designed for the target recognition of the reconstructed plankton images, and its average accuracy reaches 91.0%, which proves that the migration application of DL in the reconstruction of the digital holograms of the plankton is feasible. Moreover, based on PyQt5, the A-Unet network and the YOLOv5 network are connected together to realize the end-to-end integrated system designed for the reconstruction and recognition of plankton holograms, completing the combination of digital holography (DH) and deep learning (DL), and applying it to plankton detection. This method will promote the development of plankton detection and is of great significance for exploring the species and quantities of plankton in seawater samples. This IDRMP method will greatly enhance our understanding of marine plankton and is expected to have potential significance and attractive prospects for human beings to explore the mysteries of the ocean.

## Data Availability

Data underlying the results presented in this paper are not publicly available at this time but may be obtained from the authors upon reasonable request.

## References

[1] Reid P.C., Battle E.J.V., Batten S.D., Brander K.M. (2000). Impacts of fisheries on plankton community structure. ICES J. Mar. Sci..

[2] Rembauville M., Briggs N., Ardyna M., Uitz J., Catala P., Penkerc’h C., Poteau A., Claustre H., Blain S. (2017). Plankton assemblage estimated with BGC-Argo floats in the Southern Ocean: Implications for seasonal successions and particle export. J. Geophys. Res. Ocean..

[3] Brandini F., Michelazzo L.S., Freitas G.R., Campos G., Chuqui M., Jovane L. (2019). Carbon flow for plankton metabolism of Saco do Mamanguá Ría, Bay of Ilha Grande, a subtropical coastal environment in the South Brazil Bight. Front. Mar. Sci..

[4] De Vargas C., Audic S., Henry N., Decelle J., Mahé F., Logares R., Lara E., Berney C., Le Bescot N., Probert I. (2015). Eukaryotic plankton diversity in the sunlit ocean. Science.

[5] Karsenti E., Acinas S.G., Bork P., Bowler C., De Vargas C., Raes J., Sullivan M., Arendt D., Benzoni F., Claverie J.-M. (2011). A holistic approach to marine eco-systems biology. PLoS Biol..

[6] Huisman J., Codd G.A., Paerl H.W., Ibelings B.W., Verspagen J.M.H., Visser P.M. (2018). Cyanobacterial blooms. Nat. Rev. Microbiol..

[7] Bates N.R., Best M.H.P., Hansell D.A. (2005). Spatio-temporal distribution of dissolved inorganic carbon and net community production in the Chukchi and Beaufort Seas. Deep. Sea Res. Part II Top. Stud. Oceanogr..

[8] Bedford J., Ostle C., Johns D.G., Atkinson A., Best M., Bresnan E., Machairopoulou M., Graves C.A., Devlin M., Milligan A. (2020). Lifeform indicators reveal large-scale shifts in plankton across the North-West European shelf. Glob. Change Biol..

[9] Brunnhofer G., Bergmann A., Klug A., Kraft M. (2019). Design and validation of a holographic particle counter. Sensors.

[10] Moon I., Yi F., Javidi B. (2010). Automated three-dimensional microbial sensing and recognition using digital holography and statistical sampling. Sensors.

[11] Tan S., Wang S. (2013). An approach for sensing marine plankton using digital holographic imaging. Optik.

[12] Tan S.Z., Zhang F.Y., Huang Q.M., Wang S. (2014). Measuring and calculating geometrical parameters of marine plankton using digital laser holographic imaging. Optik.

[13] Nayak A.R., Malkiel E., McFarland M.N., Twardowski M.S., Sullivan J.S. (2021). A review of holography in the aquatic sciences: In situ characterization of particles, plankton, and small scale biophysical interactions. Front. Mar. Sci..

[14] Liu J., Du A., Wang C., Yu Z., Zheng H., Zheng B., Zhang H. Deep pyramidal residual networks for plankton image classification. Proceedings of the 2018 OCEANS-MTS/IEEE Kobe Techno-Oceans (OTO).

[15] Bachimanchi H., Midtvedt B., Midtvedt D., Selander E., Volpe G. (2022). Microplankton life histories revealed by holographic microscopy and deep learning. eLife.

[16] Cotter E., Fischell E., Lavery A. (2021). Computationally efficient processing of in situ underwater digital holograms. Limnol. Oceanogr. Methods.

[17] Latychevskaia T., Fink H.W. (2015). Practical algorithms for simulation and reconstruction of digital in-line holograms. Appl. Opt..

[18] Xu X.F., Cai L.Z., Wang Y.R., Yang X.L., Meng X.F., Dong G.Y., Shen X.X., Zhang H. (2007). Generalized phase-shifting interferometry with arbitrary unknown phase shifts: Direct wave-front reconstruction by blind phase shift extraction and its experimental verification. Appl. Phys. Lett..

[19] Luo A.J., BoozAllen J., Sullivan J., Mills S., Cukierski W. National Data Science Bowl. https://kaggle.com/competitions/datasciencebowl.2014..

[20] Zeng T., Zhu Y., Lam E.Y. (2021). Deep learning for digital holography: A review. Opt. Express.

[21] Ioffe S., Szegedy C. Batch normalization: Accelerating deep network training by reducing internal covariate shift. Proceedings of the 32nd International Conference on Machine Learning.

[22] Kingma D.P., Ba J. (2014). Adam: A method for stochastic optimization. arXiv.

[23] Yan K., Yu Y., Sun T., Asundi A., Kemao Q. (2020). Wrapped phase denoising using convolutional neural networks. Opt. Lasers Eng..

[24] Wang Z., Bovik A.C., Sheikh H.R., Simoncelli E.P. (2004). Image quality assessment: From error visibility to structural similarity. IEEE Trans Image Process..

[25] Redmon J., Divvala S., Girshick R., Farhadi A. You only look once: Unified, real-time object detection. Proceedings of the IEEE Conference on Computer Vision and Pattern Recognition.

[26] Ding Y., Yuan G., Zhou H., Wu H. (2025). ESF-DETR: A real-time and high-precision detection model for cigarette appearance. J. Real-Time Image Process..

[27] (2020). Jocher G, Stoken A, Borovec J, et al. ultralytics/yolov5: v3. 0. Zenodo.

[28] Padilla R., Passos W.L., Dias T.L.B., Netto S.L., da Silva E.A.B. (2021). A comparative analysis of object detection metrics with a companion open-source toolkit. Electronics.

[29] Willman J. (2021). Overview of Pyqt5. Modern PyQt: Create GUI Applications for Project Management, Computer Vision, and Data Analysis.

